# Variation in the ontogeny of sex steroid levels between latitudinal populations of the medaka

**DOI:** 10.1186/s40851-015-0032-1

**Published:** 2015-11-02

**Authors:** Maiko Kawajiri, Katsuhisa Uchida, Hiroaki Chiba, Shunsuke Moriyama, Kazunori Yamahira

**Affiliations:** Tropical Biosphere Research Center, University of the Ryukyus, Okinawa, 903-0213 Japan; Faculty of Agriculture, University of Miyazaki, Miyazaki, 889-2192 Japan; School of Marine Biosciences, Kitasato University, Kanagawa, 252-0373 Japan

**Keywords:** 11-ketotestosterone, Estradiol-17β, Latitude, *Oryzias latipes*, Sexual dimorphism, Sexual selection, Testosterone, TR-FIA

## Abstract

**Introduction:**

Sex steroids mediate the expression of sexual dimorphism during ontogeny, and populations that differ in the magnitudes of sexual dimorphism may accordingly differ in the ontogenetic patterns of their sex steroid levels. The medaka, *Oryzias latipes* species complex, shows geographic variation in the magnitude of sexual dimorphism with respect to the lengths of their anal and dorsal fins; dimorphism is greater in low-latitude populations than in high-latitude populations. However, sexual differences in the ontogenetic dynamics of sex steroids, and its interpopulation variation, have not been examined.

**Results:**

We measured testosterone (T), estradiol-17β (E2), and 11-ketotestosterone (11-KT) concentrations throughout ontogeny of laboratory-reared fish from two latitudinal populations: Aomori (northern) and Okinawa (southern). In both populations, the levels of all three steroids were high during early ontogenetic stages and decreased with growth. After reaching about 15 mm in standard length, when sexual dimorphisms in fin lengths became apparent, steroid levels increased and tended to plateau. Sexual differences in the steroid levels were observed only in the later ontogenetic stages; T and 11-KT levels were higher in males, while E2 levels were higher in females. Accordingly, interpopulation differences also became clearer; the southern fish tended to show higher T levels and lower E2 levels than the northern fish.

**Conclusions:**

The ontogenetic patterns of sex steroid levels paralleled the ontogeny of anal and dorsal fins in the two latitudinal populations, suggesting that interpopulation variation in the degree of sexual dimorphisms in fin lengths is mediated by sex steroid-dependent regulation of fin elongation.

## Introduction

Sexual dimorphism is widespread throughout the animal kingdom [1, 2] and arises where selection favors different phenotypic optima between the sexes. However, because genomic differences between males and females are limited, genes involved in development are faced with intralocus sexual conflicts [3, 4]. In vertebrates, one of the main mechanisms for resolving intralocus sexual conflicts is sex steroid-dependent regulation of the expression of sex-specific traits [5]. One advantage of sex steroid-mediated regulation is that the expression of sexual dimorphism can also be developmentally regulated. The titers of sex steroids generally increase at the onset of reproductive maturation in vertebrates. Developmental stage-specific expression of sexual dimorphism may be favored when, for example, sexual ornaments increase mating success in adults, but reduce juvenile survival rates [6, 7].

In many fishes, the median fins show sexual dimorphism in size and shape; e.g., the elongated caudal fins in males of the swordtail fish *Xiphophorus* [8, 9]. In some taxa, the anal fins have male-specific appendages; e.g., the gonopodium used for copulation in the male mosquito fish *Gambusia* [10, 11]. The medaka, *Oryzias latipes* species complex, also shows sexual dimorphism in the length of median fins; i.e., the anal and dorsal fins are longer in adult males than in adult females [12, 13]. Developmental stage-specific expression of sexual dimorphism is well documented in the medaka; in males, the elongation rate of the median fins with respect to standard length (SL) increases when the fish reaches about 15 mm SL, indicating development of secondary sexual characteristics [14, 15]. In females, in contrast, there is no change in the rate of fin elongation throughout ontogeny, resulting in the sexual dimorphism in adult fin lengths. However, it is unclear how the sex-specific ontogenetic patterns of fin elongation are developmentally regulated by sex steroids.

Moreover, the magnitudes of sexual dimorphism and male-specific appendages may differ between closely related species and even between populations of the same species inhabiting different habitats. For example, in *Xiphophorus* there is considerable variation among species in the patterns of elongation of the male caudal fin rays [16, 17]. Populations inhabiting contrasting environments exhibit geographical variation in sexual dimorphism in guppies [18] and sticklebacks [19, 20]. In the medaka also, geographic variation in the magnitudes of sexual dimorphisms in fin morphologies has been reported [14, 21, 22]. Generally, the anal and dorsal fins of mature males in low-latitude populations are longer than those of higher-latitude males, resulting in greater sexual dimorphism in southern than in northern populations [14, 22]. Because sex steroids are key mediators of the expression of secondary sexual characteristics in teleosts, it is essential to investigate variation in the ontogenetic changes in sex steroid levels to obtain a better understanding of the proximate mechanisms of variation in secondary sexual characteristics [23–25].

In this study, we examine the ontogenetic changes in the levels of the sex steroids testosterone (T), estradiol-17β (E2), and 11-ketotestosterone (11-KT) in two latitudinal populations of the medaka. Fish used in experiments were obtained from the northern and southern limits of their geographic range, i.e., from Aomori and Okinawa, respectively. We measured and compared sex steroid levels from fish reared in a laboratory common environment so that any differences observed could be attributed to genetic differences. We show that sex steroid levels changed during ontogeny in a manner consistent with the sexual and interpopulation patterns in fin elongation processes. Finally, we discuss possible selection pressures that have driven the evolution of interpopulation variation in secondary sexual characteristics of this fish.

## Materials and methods

### Fish

The medaka or *Oryzias latipes* species complex is a small freshwater fish that occurs in Japan, Korea, and China [13]. Mitochondrial DNA studies have revealed that Japanese populations of the medaka consist of two genetically distinct groups: the ‘northern Japan group’ distributed along the coast of the Sea of Japan in eastern Japan, and the ‘southern Japan group’ distributed along the Pacific coast of eastern Japan and in western Japan [26]. The ‘northern Japan group’ has been recently described as northern medaka *O. sakaizumii* [27], but the two groups are not reproductively isolated [28].

The lengths of the anal and dorsal fins of male medaka exhibit geographic variation; the fins are longer with respect to body size in southern males than in northern males [14, 22]. Observations of fin development in a laboratory common environment have demonstrated that the rate of fin elongation with respect to body size is also greater in southern males, in particular after about 15 mm SL, resulting in the longer fins of southern males [14, 15].

### Rearing experiment and sampling

**Fig. 1:**
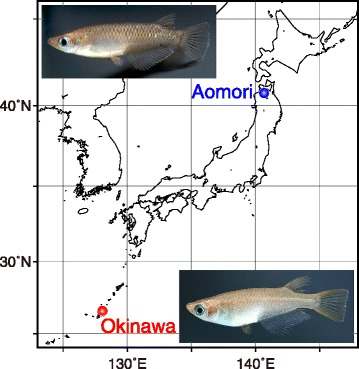
Map showing the collection sites of experimental fish and of wild medaka males. Aomori and Okinawa are located near the northern and southern limits, respectively, of the geographic range of the medaka in Japan

Wild adults were collected from Aomori (40°50′N, 140°49′E) on April 29, 2009 and from Okinawa (26°25′N, 127°48′E) on May 11, 2009 (Fig. 1). In the laboratory, we randomly chose 10 male/female pairs from the Aomori population and 21 pairs from the Okinawa population. These breeding pairs were kept in separate polypropylene containers (15 × 11 × 8 cm; water depth 5 cm) immersed within large acrylic tanks (75 × 60 × 45 cm) or in separate acrylic compartments (15 × 15 × 20 cm; water depth 17.5 mm) within the acrylic tanks. The acrylic tanks were maintained at 27 ± 0.1 °C under a photoperiod of 14 L:10D.

Newly fertilized eggs were collected from the breeding pairs, and the eggs laid on a single day by each population were pooled (50–170 eggs in total) and transferred to a polypropylene bucket (top diameter 24 cm, bottom diameter 20 cm, height 20 cm, and water depth 15 cm) immersed within FRP (fiberglass-reinforced plastic) tanks (121 × 60 × 36 cm) maintained at 26 ± 0.1 °C under the 14 L:10D photoperiod. Hatched individuals were fed daily with newly hatched *Artemia franciscana* nauplii (Silver Grade Argentemia, lot number BS1304A, Argent Chemical Laboratories, Redmond, WA, USA) and dry food (Hikari Tropical-Fancy Guppy, lot number A.09 2012.03, Kyorin Co., Ltd., Hyogo, Japan) three times per day. Eight and three replicates were conducted for the Aomori and Okinawa populations, respectively.

During the course of rearing, 2–7 individuals were randomly sampled from each bucket at intervals of 8–49 days, depending on developmental stage, until their SL averaged about 25 mm. Sampling commenced 27 days post-fertilization, when the average SL of the individuals was about 10 mm. Each individual was photographed with a digital camera (DFC320, Leica Camera AG, Solms, Germany) attached to a stereomicroscope (MZ75, Leica) or a digital camera (Coolpix 4500, Nikon Corporation, Tokyo, Japan), and SL and anal fin length (the length of the fourth anal fin ray) were later measured using Illustrator (ver. 10.0.3, Adobe Systems Inc., San Jose, CA, USA) with a plug-in software, BPT-Pro2 (Baby Universe Inc., Kanagawa, Japan). After photographing, the caudal fin of each individual was cut off and preserved in 99 % ethanol for genotypic sexing (see below). The rest of the body was preserved at −80 °C for measurements of testosterone (T) and estradiol-17β (E2).

We determined the sex of each fish as indicated by its secondary sexual characteristics [13] if the individual was large enough. For smaller individuals, the genotypic sex (XY or XX) was determined using the preserved caudal fin. Genotypic sexing was carried out on the male-determination gene *DMY* using PCR on DNA extracted from the caudal fin. We followed previously described PCR procedures [29, 30].

Before we were able to measure 11-KT on these samples, they were lost in the tsunami that struck the School of Marine Biosciences, Kitasato University, in Iwate Prefecture on March 11, 2011. Therefore, for measurement of 11-KT, we used other fish, the progeny of wild medaka collected from Aomori on June 20, 2010 and from Okinawa on July 13, 2010. Thirteen Aomori pairs and 11 Okinawa pairs from the 2010 collections were crossed as described above. Newly fertilized eggs were transferred to a polypropylene bucket (23 cm inside diameter; 20 cm height; water depth 15 cm) immersed in the temperature- and photoperiod-controlled FRP tanks. Hatched individuals were fed daily with newly hatched *A. franciscana* nauplii (Silver Grade Argentemia, lot number BS0706B) and dry food (Hikari Tropical-Fancy Guppy) three times per day. When they reached approximately 15 mm SL, 10 males and 10 females, each of which had been sexed by genotyping *DMY* as described above, were randomly chosen from the Aomori bucket and frozen for 11-KT measurement after photographing. Similarly, eight males and four females were sampled from the Okinawa bucket. The remaining individuals were transferred to glass tanks (19 × 19 × 19 cm; water depth 15 cm) immersed in the FRP tanks, separately for each population. These were kept until their SL reached about 25 mm, when 10 males and 10 females were randomly sampled, photographed, and frozen.

All procedures above were conducted according to Ethical Guidelines for Animal Experiments of University of the Ryukyus.

### Steroid hormone measurement

Steroid hormones were extracted from the frozen tissues according to the procedures described previously [31, 32]. Briefly, we cut off the caudal region of the body posterior to the vent from each frozen sample. After weighing, each tissue was homogenized in 1.0 ml phosphate-buffered saline and centrifuged at 15,000 rpm at 4 °C for 5 min. Supernatants were extracted twice with 4.0 ml of diethyl ether and the ether layers were dried and resuspended in 200 μl of assay buffer (50 mM Tris, 100 mM NaCl, 0.1 % BSA, 0.05 % NaN_3_, 0.01 % Tween 40, 0.02 mM DTPA, pH 7.75). The quantities of T, E2 and 11KT were measured by time-resolved fluorescence immunoassay (TR-FIA) [33]. Cross-reactivity of the antibodies used in the TR-FIA against the chemically similar steroids was as follows: for anti-testosterone antibody with E2 <0.01 %, progesterone <0.01 %, 17α-hydroxyprogesterone <0.01 %, cortisol <0.01 %, 11-ketotestosterone <1 %; for the anti-E2 antibody with testosterone 0.1 %, estrone 5 %, estriol 0.25 %, cortisol <0.01 %; for anti-11-ketotestosterone with testosterone 12 %, dihydrotestosterone 1.5 %, 4-androstenedione 0.5 %, and 17α-hydroxyprogesterone 0.1 % (H. Chiba, unpublished data).

### Statistical analysis

To visualize the anal fin elongation, anal fin length of each individual was plotted against SL, and a cubic spline curve was fitted using the *gam* function in the *mgcv* package (http://cran.r-project.org/web/packages/mgcv) in R (ver. 2.13.0, The R Project for Statistical Computing, 2011, www.r-project.org/) separately for each sex.

Similarly, to visualize the developmental changes of sex steroid concentrations, the quantities of T, E2, and 11-KT per unit tissue weight were plotted against SL, and a cubic spline curve was fitted separately for each sex. For analysis of T and E2 levels, we defined juvenile and adult stages as before and after 15 mm SL, respectively, because our previous study showed that secondary sexual characteristics appear at about 15 mm SL [14, 15]. However, for 11-KT analysis, we regarded individuals sampled at 15 and 25 mm SL as juveniles and adults, respectively. Sexual and interpopulation differences in hormone levels of juvenile and adult stages were tested separately using a generalized linear model (GLM) with a normal error distribution, where SL, Sex, and Population were included as explanatory variables with fixed effects. Females and Aomori were treated as the reference levels for the parameters Sex and Population, respectively. GLMs were conducted using the *glm* function in R.

## Results

**Fig. 2:**
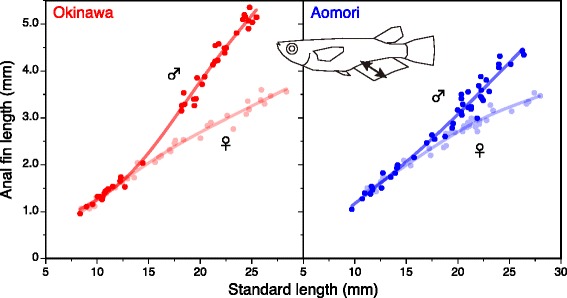
Ontogenetic changes in anal fin length in laboratory-reared individuals from two latitudinal populations. A cubic spline curve was fitted separately for each sex

Anal fin length increased with increasing body size. In males, the rate of anal fin elongation clearly increased at approximately 15 mm SL, while there was no such a change in fin elongation rates in females (Fig. 2). The acceleration of fin elongation rates was greater in Okinawa males than in Aomori males, resulting in greater sexual dimorphism in anal fin length in the Okinawa population (Fig. 2). These sexual and interpopulation patterns in fin elongation processes almost completely coincide with those obtained in the previous studies [14, 15].

**Fig. 3:**
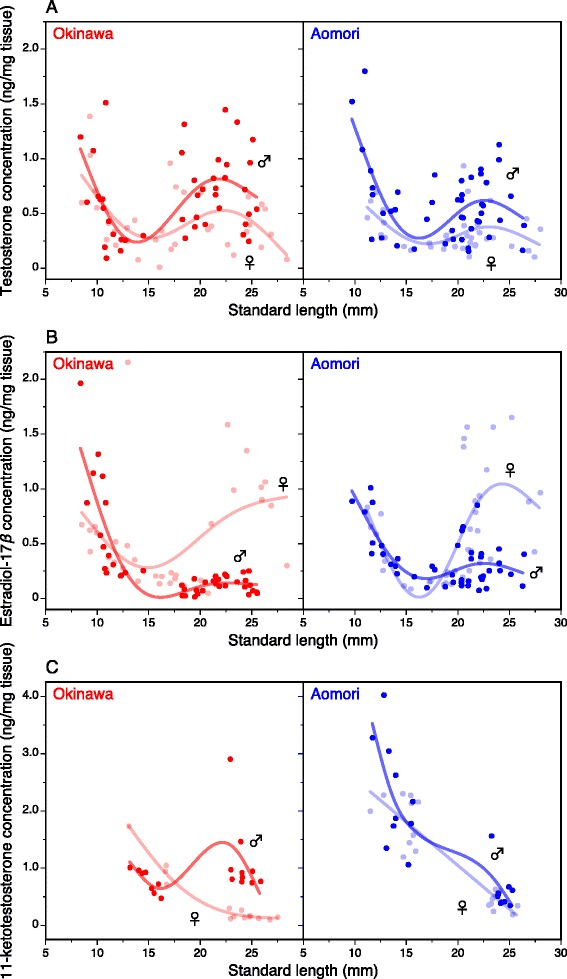
Ontogenetic changes in sex steroid concentrations in laboratory-reared individuals from two latitudinal populations. (**a**) Testosterone, (**b**) estradiol-17β, and (**c**) 11-ketotestosterone concentrations. A cubic spline curve was fitted separately for each sex

In both males and females, testosterone (T) concentrations per unit tissue weight were high when the fish were small and decreased with increasing body size up to 15 mm SL (Fig. 3a), supported by a significant effect of SL on T level at the juvenile stage (Table 1A). However, the effect of sex on T level was not significant (Table 1A), indicating no sexual differences in T level at the juvenile stage (Fig. 3a). The effect of population was significant (Table 1A), probably because T concentrations bottomed out earlier in Okinawa juveniles than in Aomori juveniles (Fig. 3a). At SL >15 mm, T concentrations increased and reached a plateau (Fig. 3a). The T concentration at the adult stage tended to be higher in males than in females, supported by a significant effect of sex (Table 2A). Moreover, the effect of population on adult T concentration was also significant (Table 2A), indicating that Okinawa males and females have higher T levels than Aomori males and females, respectively (Fig. 3a).

**Table 1:** Results of the GLM of the effects of SL, sex, and population on sex steroid levels during the juvenile stage

Parameter	Estimate	Std. error	*t* value	*P*
A. Testosterone				
Intercept	2.61777	0.38081	6.874	<0.0001
SL	−0.16602	0.02956	−5.617	<0.0001
Sex	0.10278	0.08649	1.188	0.2406
Population	−0.30077	0.09420	−3.193	0.0025
B. Estradiol-17β				
Intercept	2.11597	0.48308	4.380	<0.0001
SL	−0.12957	0.03738	−3.467	0.0012
Sex	0.03758	0.10858	0.346	0.7308
Population	−0.08264	0.12036	−0.687	0.4958
C. 11-ketotestosterone				
Intercept	5.25412	1.17611	4.467	<0.0001
SL	−0.22068	0.07901	−2.793	0.0093
Sex	−0.02898	0.21527	−0.135	0.8939
Population	−0.99220	0.22396	−4.430	0.0001

Females and Aomori are the reference levels for the parameters Sex and Population, respectively

**Table 2:** Results of the GLM of the effects of SL, sex, and population on sex steroid levels during the adult stage

Parameter	Estimate	Std. error	*t* value	*P*
A. Testosterone				
Intercept	0.220863	0.186066	1.187	0.2379
SL	0.002920	0.008484	0.344	0.7314
Sex	0.271329	0.050003	5.426	<0.0001
Population	0.147938	0.050347	2.938	0.0040
B. Estradiol-17β				
Intercept	−0.44271	0.22288	−1.986	0.0496
SL	0.05191	0.01028	5.052	<0.0001
Sex	−0.40672	0.06039	−6.735	<0.0001
Population	−0.14185	0.06050	−2.345	0.0209
C. 11-ketotestosterone				
Intercept	3.6949	1.4588	2.533	0.0158
SL	−0.1433	0.0601	−2.384	0.0225
Sex	0.5498	0.1276	4.311	<0.0001
Population	0.1652	0.1281	1.289	0.2056

Females and Aomori are the reference levels for the parameters Sex and Population, respectively

Estradiol-17ß (E2) concentration also significantly decreased with increasing SL during the juvenile stage in both populations (Fig. 3b, Table 1B). However, the effects of sex and population on E2 level were not significant (Table 1B), indicating no sexual and interpopulation differences in E2 level at the juvenile stage (Fig. 3b). After 15 mm SL, E2 levels of females increased and tended to reach a plateau in both populations but the peak of the plateau was much higher in females than in males (Fig. 3b). This sexual difference in ontogenetic dynamics of E2 levels was supported by a significant effect of sex (Table 2B). The effect of population on E2 concentrations at the adult stage was also significant (Table 2B), indicating that E2 levels are higher in Aomori males and females than in Okinawa males and females, respectively (Fig. 3b).

The concentration of 11-ketotestosterone (11-KT) also significantly decreased with increasing SL at the juvenile stage (Fig. 3c, Table 1C). The effect of sex on 11-KT level was not significant (Table 1C), indicating no sexual differences in the 11-KT dynamics during the juvenile stage (Fig. 3c). The effect of population was significant (Table 1C), because Okinawa juveniles showed lower 11-KT concentrations than Aomori juveniles, although the small number of samples before 15 mm SL precluded detection of ontogenetic patterns. At the adult stage, the effect of sex was significant (Table 2C), probably because 11-KT concentration was clearly sexually dimorphic in the Okinawa population, i.e., males had a higher average concentration than females (Fig. 3c).

## Discussion

### Sex-specific ontogenetic dynamics of sex steroid levels

We found that T, E2, and 11-KT levels are all high in early ontogenetic stages and decreased with their growth. Similar ontogenetic patterns of sex steroid levels during early developmental stages have also been reported in other fish species, including tilapia [34] and coho salmon [35]. These observations suggest that the high sex steroid levels in early developmental stages are involved in organogenesis rather than in the expression of sexual dimorphism. There were no significant sexual differences in the steroid levels in juveniles, which supports this view.

Significant differences in sex steroid levels between sexes were observed only at later ontogenetic stages. In vertebrates, most sexually dimorphic traits develop after gonadal maturation under the influence of the sex-specific hormones [5]. Adult sexual dimorphisms in the medaka at later ontogenetic stages may also be mediated by differences in sex steroid levels. In males, T concentration decreased until about 15 mm SL, and increased after that. A similar pattern was observed in 11-KT, although the sample size was larger at the juvenile stage. This pattern of fluctuation of T levels, and possibly also of 11-KT levels, is consistent with the developmental timing of sexually dimorphic traits; the rate of elongation of the anal and dorsal fins clearly increased in males at about 15 mm SL [14, 15]. In the medaka, gonadal differentiation is known to occur at about 5.5 mm SL, and testis and ovary continue to develop throughout their ontogeny [13]. Spermatocytes can be observed in testes at about 20–23 mm SL, suggesting that T and 11-KT concentrations begin to increase before they are able to reproduce. In contrast, development of oocytes in the ovaries starts earlier than spermatocyte development but the oviducts do not open until about 23 mm SL [13]. E2 concentration clearly increased in females at around 15 mm SL and therefore may be involved in oogenesis.

Some studies have reported changes in androgen-dependent gene expression for elongation of fins as sexually dimorphic traits. For example, androgen-dependent *sonic hedgehog* (s*hh*) expression is required for anal-fin outgrowth, leading to the formation of a genital appendage, the gonopodium, in *Gambusia affinis* [10]. In male swordtails (*Xiphophorus*), testosterone treatment activates a gene network that positively controls the expression of *muscle segment homeobox C* (*msxC*) through signaling by *fibroblast growth factor receptor 1* (*fgfr1*) during the development of the swords and gonopodial rays [8, 9]. In the medaka, the development of papillary processes on the anal-fin rays is promoted by androgen-dependent augmentation of *bone morphogenetic protein 7* (*bmp7*) and *lymphoid enhancer-binding factor-1* (*lef1*) [36]. However, androgen- and/or estrogen-dependent expression of genes that mediate fin elongation as sexually dimorphic traits is largely unknown in the medaka.

### Interpopulation variation in the ontogeny of sex steroid levels

Sex steroid levels at the adult stage were different between the populations. The average T concentration at the adult stage was higher in Okinawa males than in Aomori males. The 11-KT concentration may also have been higher in Okinawa males although the difference was not significant because of the low number of samples. In teleost fish, sexually dimorphic traits, such as modified or extended fins, are induced by androgen. Adding exogenous androgen induces fin elongation as a masculine trait in female *Gambusia* [37] and in some normally swordless platies (*Xiphophorus* spp.) [38]. The more conspicuous secondary sex characteristics in the Okinawa male medaka than in Aomori males are also probably caused by higher concentrations of T and 11-KT. In addition, the average E2 concentration at the adult stage was higher in Aomori males than in Okinawa males. Estrogen tends to induce feminine traits and inhibit masculine traits. In the medaka, for example, male-specific development of chromatophores associated with nuptial coloration is inhibited by estrogen [13, 39, 40]. The high levels of endogenous E2 observed in Aomori males may inhibit their masculine traits, increasing the difference in the degree of sexual dimorphism between the populations.

**Fig. 4:**
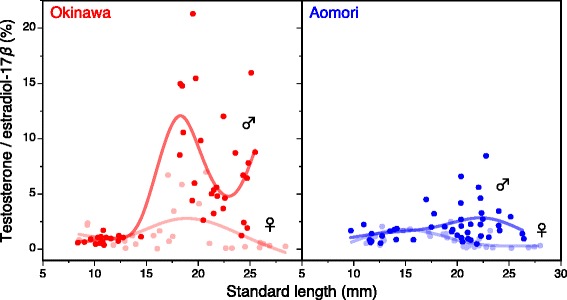
Ontogenetic changes in the ratio of testosterone to estradiol-17β levels in laboratory-reared individuals from two latitudinal populations. The ratio of T to E2 per unit tissue was calculated for each individual and plotted against SL. A cubic spline curve was fitted separately for each sex, using the *gam* function in the *mgcv* package in R ver. 2.13.0

Correspondingly, we found that the ratio of T to E2 levels changed during ontogeny in a manner consistent with the sexual and interpopulation patterns in fin elongation processes; the ratio increased after about 15 mm SL more conspicuously in Okinawa males than in Aomori males, while no conspicuous ontogenetic change was observed in females (Fig. 4). This pattern suggests antagonistic actions between androgen and estrogen in the expression of masculinity, but the molecular mechanisms this putative antagonism remain unclear.

Recently, quantitative trait loci (QTL) mappings were performed to investigate the genetic architecture of the variation in the ontogeny of sexual dimorphism in fin lengths between Aomori and Okinawa populations [15]. The results revealed that few QTL have consistent effects throughout ontogenetic stages, and that the majority of QTL change the magnitudes and directions of effects on fin elongation rates during ontogeny, indicating interpopulation variation in size-specific gene expressions. Interestingly, many genes involved in steroid metabolism, such as *cytochrome P450* (*CYP*) genes, were found near the QTL [15], suggesting that variation in the size-specific expression of these steroid metabolism genes results in the interpopulation variation in sexual dimorphism in fin lengths. It has also been shown that polymorphisms in *CYP* genes are associated with variation in sexual dimorphism in the anal fin morphology between Japanese and Korean populations; high-activity alleles enhance sexual difference in anal fin shapes [21], supporting that *CYP* genes are responsible for the interpopulation variation in the ontogeny of sex steroid levels and resultant variation in fin lengths.

### Selection on the ontogenetic dynamics of sex steroid levels

Hormones have pleiotropic effects on phenotypic traits. For example, androgens are known to increase the expression of male-specific appendages or ornaments but they also promote courtship behaviors and territorial aggressions [41–44]. In support of this, it has been demonstrated that male medaka from lower-latitude populations fight each other more frequently than do males from higher-latitude populations and that lower-latitude males also tend to court females more frequently [21]. Theoretically, such male-specific appendages and mating behaviors are thought to evolve through sexual selection [2, 45]. Indeed, it has been reported in the medaka that males with longer fins and stronger eagerness in courtship are preferred by females over males with shorter fins and weaker eagerness in courtship [21, 46, 47]. The more conspicuous secondary sexual characteristics and more active mating behaviors in Okinawa males than in Aomori males, which are probably mediated by high endogenous androgen levels, therefore implies that sexual selection pressures are stronger in Okinawa than in Aomori. In contrast, Katsumura and colleagues have argued that the reduced sexual dimorphism in Korean populations may have evolved as a by-product of adaptation to water pollution [21]. However, there is no circumstantial evidence that water pollution has been severer in Aomori than in Okinawa, which leads us to support our own view that variation in sexual selection pressures between the populations is the primary cause for the interpopulation variation in sexual dimorphisms and male mating behaviors.

Nevertheless, it is unclear why sexual selection pressures are stronger at lower latitudes. Theories predict that the strength of sexual selection is determined by the operational sex ratio (OSR) within populations; biased OSRs cause strong sexual selection because the more abundant sex, usually males, compete for available partners [48, 49]. We stress that OSRs must be compared between high- and low-latitude wild populations by population ecological approaches to explore the reasons behind the evolution of latitudinal variation in mating behavior in this fish.

## Conclusions

The observed ontogenetic patterns of sex steroid levels parallel the ontogeny of the anal and dorsal fins in two latitudinal populations of the medaka, suggesting that interpopulation variation in the degree of sexual dimorphism in this fish is mediated by sex steroid-dependent gene expression. This study provides a starting point for ‘evo-devo-eco’ studies aimed at elucidation of the molecular and genetic mechanisms involved in the variation in the ontogeny of sex steroid levels and determination of the selective forces driving the evolution of sexual dimorphism in the wild.
